# Predictive Models for the Characterization of Internal Defects in Additive Materials from Active Thermography Sequences Supported by Machine Learning Methods

**DOI:** 10.3390/s20143982

**Published:** 2020-07-17

**Authors:** Manuel Rodríguez-Martín, José G. Fueyo, Diego Gonzalez-Aguilera, Francisco J. Madruga, Roberto García-Martín, Ángel Luis Muñóz, Javier Pisonero

**Affiliations:** 1 Department of Mechanical Engineering, Universidad de Salamanca, 37008 Salamanca, Spain; ingmanuel@usal.es (M.R.-M.); fueyo@usal.es (J.G.F.); toles@usal.es (R.G.-M.); 2 Department of Technology, Universidad Católica de Ávila, 05005 Ávila, Spain; 3 Department of Cartographic and Land Engineering, Universidad de Salamanca, 05003 Ávila, Spain; almuni@usal.es (Á.L.M.); j_pisonero@usal.es (J.P.); 4 Photonics Engineering Group, CIBER-BBN and IDIVAL, Universidad de Cantabria, 39005 Santander, Cantabria, Spain; francisco.madruga@unican.es

**Keywords:** active thermography (AT), finite element method (FEM), non-destructive testing (NDT), quality assessment (QA), machine learning (ML), additive materials (AM)

## Abstract

The present article addresses a generation of predictive models that assesses the thickness and length of internal defects in additive manufacturing materials. These modes use data from the application of active transient thermography numerical simulation. In this manner, the raised procedure is an ad-hoc hybrid method that integrates finite element simulation and machine learning models using different predictive feature sets and characteristics (i.e., regression, Gaussian regression, support vector machines, multilayer perceptron, and random forest). The performance results for each model were statistically analyzed, evaluated, and compared in terms of predictive performance, processing time, and outlier sensibility to facilitate the choice of a predictive method to obtain the thickness and length of an internal defect from thermographic monitoring. The best model to predictdefect thickness with six thermal features was interaction linear regression. To make predictive models for defect length and thickness, the best model was Gaussian process regression. However, models such as support vector machines also had significative advantages in terms of processing time and adequate performance for certain feature sets. In this way, the results showed that the predictive capability of some types of algorithms could allow for the detection and measurement of internal defects in materials produced by additive manufacturing using active thermography as a non-destructive test.

## 1. Introduction

Every industrial manufacturing process aims for the highest possible quality. Generally speaking, decreases in quality standards are linked to a wide range of defects that are inherent to manufacturing processes. These defects may be internal and may lead to failure and collapse of those structures, devices, or machines with additive-manufactured functional parts. Dealing with defects implies previous actions that detect and repairs parts in which they appeared or dismissed them, especially if the repair costs exceeded the manufacturing costs of new parts. Quality requirements are more critical in additive manufacturing (AM), which allows for the production of customized elements, even with complex geometries with no restrictions provoked by traditional manufacturing processes [1].

There are different defect detection methods based on destructive or non-destructive testing (NDT). Thermography is an NDT that can be classified by the type of information obtained (qualitative and quantitative) and also by the method used: active thermography (AT) or passive thermography (PT). ATs working principle is that defects and other types of discontinuities in materials tructure can alter a specimen’s diffusivity and cause heat flow alterations [2]. In this work, we focus on AT and the detection of internal defects [3], as well as their dimensional analysis [4].

Physical properties, such as the temperature [5] or heating-cooling rate obtained from AT [6], can be used as predictive parameters whene stimating the depth of cracks in steel. In fact, in Rodríguez-Martin et al. [6], a pixelwise algorithm for time derivative of temperature (PATDT) was developed to predict geometric features of the crack in steel welds from a sequence of thermograms using AT.

Numerical methods are useful to complement and optimize AT cost and efficiency [7]. Using them, the equation of thermal conductivity [8] can be numerically solved via sophisticated simulation tools. 3D modelling is also a useful tool to provide information about the influence of different factors, such as changes in the dimensions of defects and their depths [9].

Different authors have applied the finite element method (FEM) to investigate the heat transfer phenomenon during thermographic inspections using different codes and solutions like Ansys [9,10] or Abaqus [11]. However, the application of numerical methods normally entails the assumption of simplifications that may impact the interpretation of physical phenomenon, such as the uniform nature of heating applied or the variability of density, among others. In turn, 3D modelling and simulation methods allow for the generation of ideal surfaces under which geometrical conditions may differ from the real ones. Carvalho et al. [7] apply a model based on FEM simulation to solve the heat transient problem, while other authors apply a surface flux [10,12,13,14].

The datasets obtained using FEM can be useful to estimate parameters and thus to train predictive models, allowing for the estimation of a geometrical feature of the defect from thermal features. Within the machine learning (ML) approaches, regression learners study the relationship between one or more explanatory random variables and their responses [15]. Specifically, the artificial neural network (ANN) has been applied for regressions in various investigations with thermography to estimate the depth of the defects [4,16] or for biomedical applications [17]. ANN can be applied using visualization approaches that provide information about its behavior and structure [18]. ANN and support vector machines (SVM) models have also been applied for coating thickness estimation [19]. Regression learner using the Gaussian process regression model (GPR) has been applied to results of different computational fluid-dynamic simulations, interpolating the positions where experimental data were unknown [20]. Thermography has also been combined with deep learning (DL) strategies to detect cracks in steel [21].

Initially, AM was used to manufacture prototypes, but today the production of final parts for engineering applications is demanded and therefore technical plastics take on special relevance [22]. Nylon stands out with specific properties: a semi-crystal polyamide polymer; very low specific weight; excellent tensile strength and elastic recovery; toughness; resistance to bending and wear; and a good surface finish [23,24,25]. This material is usually processed using powder-bed laser sintering [26] but we can find more accurate and flexible processes such as inkjet-based manufacturing [22]. It should be highlighted that Nylon can be recycled for later use in the additive manufacturing process as a component for forming enhanced physical mechanical composites. This is helpful to minimize the environmental impact of non-biodegradable polymers [24].

In this article, a FEM simulation configuration is established using the physical properties of Nylon PA-12. Different types of models were designed and trained using different feature sets in order to establish the more efficient model and the thermal features needed to predict the geometrical features as response. Since different thermal features are relevant in the heat transfer process, it is convenient to know those that have a greater influence on the prediction of the defect geometry which can be used as input for the prediction model. These models could serve to design intelligent, automated, and non-destructive inspection protocols of additive-manufactured parts using active thermography.

Performance results for several generated multiparameter models are scientifically compared. In this way, a predictive technique based on the last advances in ML is proposed for the estimation of the geometric parameters of internal defects, using the thermal properties acquired with AT.

## 2. Materials and Methods

**Figure 1:**
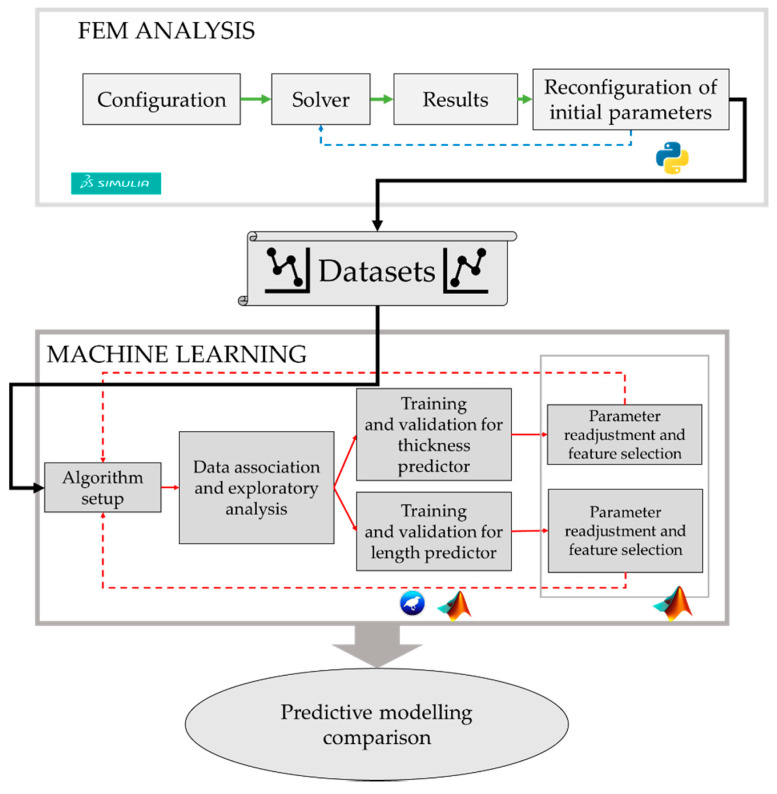
Workflow for the hybrid methodology applied.

An ad-hoc hybrid strategy that integrates FEM and ML was designed to address this research. The two phases are described in the workflow outlined in Figure 1.

### 2.1. Numerical Model Design

Additive manufacturing (AM) procedures use different techniques for material deposition. Depending on these techniques, pores can be confused with small defects and appear, causing variations in the thermomechanical behavior in the different points and working directions. This problem was studied in laminated object manufacturing (LOM) [27] and fused filament fabrication (FFF) [28]. However, addressing this issue would imply a study of material properties after deposition at the mesostructural level and the preparation of a FEM model capable of simulating it. Although the study of this problem could be extremely interesting, it would enormously complicate this work. Thus, in this study, a simpler approach was carried out in which the macrostructural thermomechanical behavior of the material was considered as continuum and isotropic. This approach was previously considered in several works [7,12,14].

**Figure 2:**
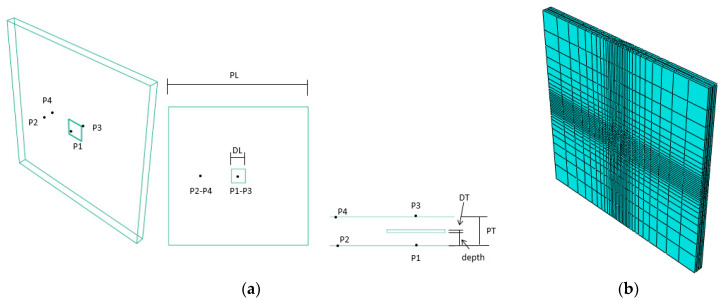
(**a**) Geometry of the model and the defect and location of the points used in the thermal study. (**b**) Biased mesh with greater mesh density close to the defect area. *PL* and*DL* refer to the length of the plate and defect, respectively, being *PL* = 0.1 m and *DL* = 0.01 m, whereas *PT* and*DT* refer to the thickness of the plate and defect, respectively, being *PT* = 0.005 m and *DT* = 0.0005 m.

A FEM model was designed to study the effect that an internal defect (e.g., a hole-like) provokes in the heat flux and the temperature distribution. The geometry and the principal dimensions of the FEM model proposed in this work can be seen in Figure 2b. In addition, four points, *P1*–*P2* and *P3*–*P4*, were located on the upper and lower surfaces, respectively, in order to study the evolution of temperatures through time. *P1* and *P3* are close to the defect, while *P2* and *P4* are far from it. The distance between *P1*–*P2* and *P3*–*P4* is 0.025 mm. The comparison between these points allows us to see the effect of the defect together with its superficial temperature distribution, using different thermal loads applied to the model. Considering that the model was prepared with a small thickness, it was possible to study the effect of the defect in the upper surface (reflection case) and lower surface (transmission case). The reflection case studies the temperature trace in the upper surface, where the heat excitation is applied. For its part, the transmission case studies the temperature trace in the lower surface, i.e., in the opposite side where the heat excitation is applied. This model was used to study the effect of the principal thermal properties (i.e., conductivity, specific heat, density, film coefficient, and emissivity coefficient) on both surfaces. The properties of the material were those corresponding to a polymeric material Nylon PA-12, a widely used material in 3D printing. All these material properties, the geometry and the heat process were proposed following [7] and can be seen in Table 1.

The model was subjected to a heating process (heating-step) in its upper face from 24 °C to 120 °C through a linear ramp for 20 s. Once the highest temperature was reached, the heat source moved away and the model started to exchange heat with the external environment through convection and radiation heat transfer processes (cooling-step). The studied values of this interaction can be seen in Table 1.

Finally, the model was meshed with DC3D8 for heat transfer 3D 8-node linear isoparametric elements using the commercially FEM software Abaqus2019^®^ [11,12]. A biased, non-uniform meshing was defined to increase the density of elements in defective areas, improving the precision of data, and reducing the density of elements in the background area. The number of elements was reduced to 25% of the number of elements corresponding to a uniform mesh, maintaining the same precision in the areas close to the defect (Figure 2). To complete the meshing design, different convergence analyses were conducted in order to obtain a mesh size, which can give accurate results in the defect areas, without penalizing considerably the time needed to compute the models. A size element of 1 mm was considered precise enough without penalizing the computational cost. Finally, each model had 5020 elements.

Several command lines were added, using Python language to the file created by Abaqus. These command lines were programmed to obtain temporal evolutions of the temperatures in points *P1* to *P4*. More command lines were used to plot contrast curves based on temperature versustime between points *P1*–*P2* and *P3*–*P4*. The higher this contrast, the easier it is to detect a defect, as well as its size and location.

After all these steps were completed, the obtained results were used to apply ML techniques that allowed us to estimate the geometrical features of the defects using AT data.

### 2.2. Machine Learning Modelling

Different regression learners were applied and trained to compare their performances. The same model type was trained using different sets of features and different k-fold validations and/or hyperparameters in order to obtain the best performance setup.

MATLAB © [29] was used to train the next model types: linear regression, GPR, and SVM, while the open source software, Weka [30], was applied to train the random forest (RF) and multilayer perceptron (MLP) models. All the models were trained considering different features frames and parameters. The results of unsuccessful models were not reported, although some of them were indicated in Appendix A. The different predictive model typologies used are widely defined in the literature, yet in order to contextualize the raised research, a brief description of each of them is given below.

#### 2.2.1. Linear Regression Model

Linear regression models are predictive algorithms which are easy to interpret and fast to predict. However, these models provide a low flexibility and their highly constrained form means that they usually have poor predictive accuracy compared to other more complex models. In this case, three different linear regression models were applied: (i) linear regression which uses a constant and linear term; (ii) interaction linear regression which applies interaction between predictors; (iii) stepwise linear regression, which analyses the significance of each variable [31]. In this work, we considered stepwise linear regression to prioritize the detection potential of the algorithm with respect to the physical significance of the statistical relationships between variables.

#### 2.2.2. Gaussian Process Regression Model (GPR)

In the last decade, the GPR model has attracted considerable attention, especially in ML approaches [32]. These methods apply non-parametric kernel functions based on probabilistic models (Bayesian inference) [20]. These non-parametric methods are usually more rigorous than the standard regression methods described above, especially for the treatment of complex and noisy non-linear functions [33] and its cross validation [34].

#### 2.2.3. Support Vector Machine

SVM are supervised learning models initially used for classification problems but also for robust regression solutions [31]. SVM are non-parametric techniques that are still affected by outliers [35]. SVM robust regression may be useful to add robust estimators based on variable weight functions [31]. The flexibility of SVM methods are due to the kernel functions (radial basis function (RBF), quadratic, cubic, or linear) [36]. In this research, the four kernel functions were used. Furthermore, for RBF, three different kernel scales were used: fine, medium, and coarse. Those prediction errors that were smaller than the threshold (*ε*) were ignored and treated as equal to zero. Epsilon mode was automatically calculated using a heuristic procedure to select the kernel scale.

#### 2.2.4. Random Forest

RF [37] is a known ensemble classifier that can be used for both classification and regression, like trees, where each tree is generated from different bootstrapped samples of training data [38], enabling many weakly-correlated classifiers form a strong classifier. RF is usually easy to implement and computationally fast, which performs well in many real-world tasks.

#### 2.2.5. Multilayer Perceptron

MLP is an ANN method that uses backpropagation to learn a multilayer perceptron to classify instances. The MLP allows to represent some smooth measurable functional relationships between the inputs (predictors features) and the outputs (responses). MLP is a distributed, information processing system massively parallel and successfully applied for the generation of models to solve non-linear problems [39,40]. The processes are based on three different layers of neurons: input layers (*N* neurons), hidden layers (*S* neurons) and output layers (*L* neurons), where each layer has a group of connected points (neurons). Each connection has a numerical weight and each neuron of the network performs as a weighted sum of its inputs and thresholds the results. The momentum rate for the backpropagation algorithm was established as 0.2 for the standard value and 0.3 for the learning rate, while nominal to binary filter was applied. Hidden layers were established as *(attributes+ classes)/2* for each test.

### 2.3. Evaluation of the Model Performance

The evaluation of the models can be implemented by assessing the difference between the observed values ($\hat{y_{j}}$) and predicted values ($y_{j}$) [20]. The performance of the regression learning models can be evaluated using classical performance results [41]. In this research, three statistical error types were obtained for each model:

- Determination of the correlation coefficient (*R*2) between observed values and predicted values (1). When it is closer to 1, the correlation between observed and predicted values will be more adjusted. A theoretical value of 1 means a perfect correlation between the observed and predicted values, which could be interpreted as a perfect prediction (graphically, this would mean that all points represented in the predicted vs. actual plot are located in the regression line).
- Mean absolute error (*MAE*): this error describes the typical magnitude of the residuals being robust to outliers (2). *MAE* was used to independently evaluate the accuracy of the model.
- Mean square error (*MSE*): this error estimation was computed considering the square of the differences, being more sensitive to outliers than *MAE*.
- Root mean square error (*RMSE*): it was calculated as the square root of the *MSE* (3). In this way, the error data was converted to the units of the variable, making the data interpretation more intuitive in the magnitude of the response.
  (1) $$R^{2}=1-\frac{\sum_{j=1}^{n}{\left(y_{j}-\hat{y_{j}}\right)}^{2}}{\sum_{j=1}^{n}{\left(y_{j}-\bar{y_{j}}\right)}^{2}}$$
  (2) $$MAE=\frac{1}{n}\sum_{j=1}^{n}\left|y_{j}-\hat{y_{j}}\right|$$
  (3) $$RMSE=\sqrt{\frac{1}{n}\sum_{j=1}^{n}{\left(y_{j}-\hat{y_{j}}\right)}^{2}}$$

Finally, the training time is a parameter that was reported for each model in order to compare the response speed of each algorithm. To this end, all the trainings of the different models were implemented in an Intel Core i7-5700HQ, 2.7 GHz CPU without parallel computing. Additionally, the distribution and morphology of the residuals was another performance model indicator evaluated.

## 3. Results

### 3.1. Simulation Results

**Figure 3:**
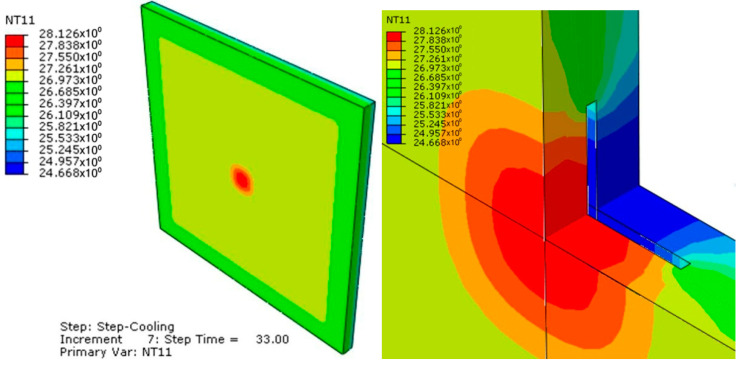
Model temperature distribution at 53 s.

Some of the calculations carried out and the results achieved in this study are shown below. With the initial values of the geometric variables, the thermal properties of the material and the thermal load curves applied, a calculation of the temperature distribution along the whole model was performed. Figure 3 shows the temperature distribution in the model at 53 s.

**Figure 4:**
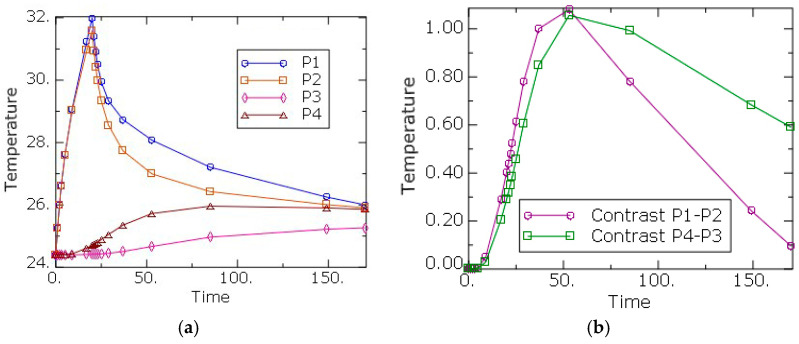
(**a**) Temperature vs. time at points *P1* to *P4*. (**b**) Temperature contrast curves *P1*–*P2* and *P4*–*P3* vs. time.

Using the script developed in Python, the variation of the temperatures through time at points *P1*–*P4* was recorded. Figure 4a shows the values of the temperatures through time at points *P1*–*P4*.

Also, the temperature contrast curves, *P1* minus *P2* and *P4* minus *P3*, through time were calculated and plotted (Figure 4b), which show the difference of temperatures between areas near and far to the defect. Contrast curve *P1*–*P2* shows how the presence of the defect affects the upper surface, the so-called reflection case, while contrast curve *P4*–*P3*, exhibits the effect of the defect on the rear surface, that is, due to the difference of transmissibility temperature between zones with defects and zones without them. It can be also observed how the maximum in the temperature curves appears at 53 s of the total time, that is, 33 s after the start of the cooling step.

The point of maximum contrast is of great interest since it would allow to determine the presence of the defect and its characteristics. Therefore, these points were used to analyze the variation of the input variables (i.e., thermal properties, size and thickness of the defect) over the upper face-reflection case (Contrast Front ($\Delta T_{F}$)) and over the lower face-transmission case (contrast rear ($\Delta T_{R}$)). The ranges of variation of the input variables established for this research are outlined in Table 1. The sets of values used in each simulation were automatically selected by the software within the thresholds indicated in this table. In this manner, two datasets first repeated the simulation 100 times and the second repeated the simulation 500 times.

**Figure 5:**
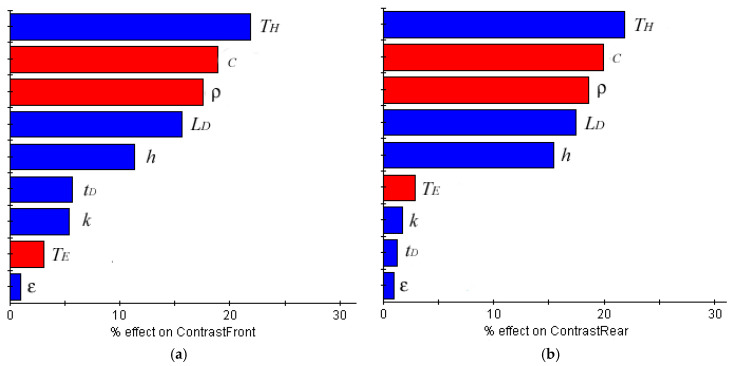
Pareto plot indicating the influence weight of each input variable in responses (**a**) “Contrast Front” and(**b**) “Contrast Rear”.

A design of experiment (DOE) study was carried out using the Latin hypercube technique with 500 points. Figure 5 shows the Pareto plot for responses “Contrast Front” and “Contrast Rear”. The size of the bars indicates the proportion in which each one of the input variables affects the variation of the output variables. The blue color indicates that the relationship is direct, while the red color indicates that is inverse, i.e., if the value of the input variable increases, the value of the output decreases and vice versa.

Figure 5 shows how the most influential variable in both cases was the maximum heating temperature ($T_{H}$). This indicates the need to carry out a good design of the thermal loading process, adjusting this temperature as much as possible. Moreover, the size of the defect ($L_{D}$) had a high weight that indicates that the magnitude of the contrast could be used to estimate the size of the defect. On the other hand, it seems significant how, in both cases, the thickness of the defect ($t_{D}$) had a low effect, especially in the “Contrast Rear” case.

**Figure 6:**
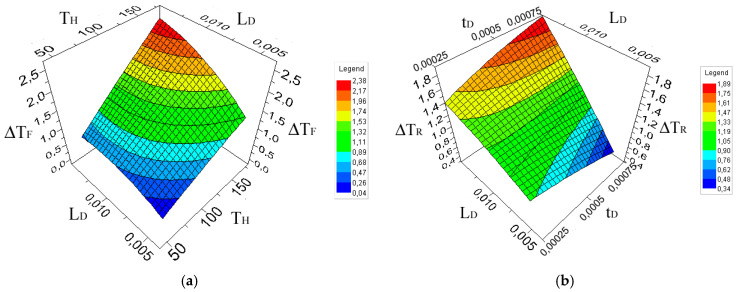
Approximation surfaces for different input and output variables combinations. (**a**) “Contrast Front”; (**b**) “Contrast Rear”.

Finally, Figure 6 shows two of the many possible approximated surfaces that can be prepared to study the variation of the output values as a function of the variation of the input values. In Figure 6b, it can be seen that the variation of “Contrast Front” with the variation of the maximum heating temperature and with the size of the defect. Since both input variables have a high effect, the surface varies almost equally in both base coordinates. Instead, in Figure 6a, the “Contrast Rear” is shown in relationship with the length and thickness of the defect. Because the thickness of the defect has a smaller effect, the approximated surface changes more along the length related coordinate.

### 3.2. Machine Learning

First, an exploratory data analysis was applied to the datasets for both the 100-value and the 500-value. This was implemented using scattering plots, which showed similar trends in the relationship of the features for the two data collections. An apparent collinearity is detected between the “Contrast Font” ($\Delta T_{F}$) and “Contrast Rear” ($\Delta T_{R}$) because both are independent with respect to the rest of the features. This phenomenon could be due to the heat transfer and the presence of the defect, which makes the difference in temperature between the defect and non-defect zones very similar in both sides of the model. However, the relationship between the two features is not rigorously linear because it has a non-constant variability. The rest of variables are independent since they are inputs for the simulation processes (Table 1). Therefore, in the following sections, different tests were implemented in order to find an adequate parsimonious model with the fewest assumptions. For the different trained models, *MAE*, *R*2, and *RMSE* were reported. The rest of parameters analyzed for each model were reported in the Appendix A in order to make easier its reading. Please note that in the finite element part, the geometric variables and the thermal properties are inputs, while the temperature contrasts $\Delta T_{F}$ and $\Delta T_{R}$ are outputs. On the other hand, this changes in the machine learning part of the study; the contrasts become $\Delta T_{F}$ and $\Delta T_{R}$ together with the thermal properties inputs, while the geometric variables length and thickness of the defect are outputs, being this last the variables to predict using the model.

#### 3.2.1. Defect Thickness Predictor

Firstly, models were trained only using the 100 sets of values to analyze what happens to a small sample size, but the predictive capacity was low. The most accurate model yielded poor prediction results (e.g., stepwise regression model was the more effective yielding *R*2 = 0.45). Then, the predictor model was calculated using the 500 sets of values of the dataset. The results for the models were trained using 500 sets of values (Table 2). The “Contrast Rear” feature had to be included in the model to get suitable results. Otherwise, the predictive performance decreased significantly and the models obtained were not adequate (maximum *R*2 = 0.35).

The best results were achieved by the following two models: stepwise regression model and interaction regression model, although the training time is extremely much longer for the stepwise, as shown in Appendix A. The best predictor was the one that used all features (*MAE* = 5.148 × 10^−5^, *R*2 = 0.79). The error obtained was acceptable considering the range and order of magnitude of the predicted variable (5 × 10^−4^ ± 50% mmin Table 1) However, when only 5 features ($k,\;h,T_{H},\Delta T_{F},\Delta T_{R}$) were considered (5 excluded), *MAE* increased by 36.71% and *R*2 was 0.63. For the thickness predictor, it was always necessary to consider “Contrast Rear” $\left(\Delta T_{R}\right)$ to obtain suitable results.

Additionally, all the models were calculated using three different k-fold validation parameter (5, 10, and 15). The results for the 10-fold validation were reported and the *MAE* of the other two k-fold’s validations, and indicated as deviation in the Appendix A. In this way, deviation values for the *MAE* were not meaningfully high. The results referred to the regression models and are reported in this section because the rest of the models (i.e., GPR, SVM, RF, and MLP) did not provide suitable results due to the small size of the dataset.

**Figure 7:**
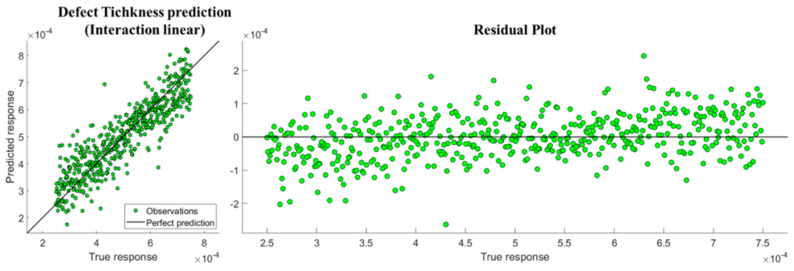
Interaction regression model for all the features (*R*2 = 0.79, *MAE* = 5.148 × 10^−5^).

An appropriate linear relationship between predicted response and observed response was observed for all the predictions. In Figure 7, this regression line is shown for the predictor model, which provides a minor MAE (Table 2). Residuals are close to a symmetrical distribution around zero.

#### 3.2.2. Defect Length Predictor

Firstly, the experiment was implemented using 100 sets of values. Unlike in the predictive thickness model, in this case, significative different results were obtained considering or not considering the “Contrast Rear” feature ($\Delta T_{R}$), so this aspect allowed us to compare the model performance when $\Delta T_{R}$ wasconsidered or excluded. Consequently, the results for the two configurations are reported (Table 3) and the different predictive features are removed in order to analyze the model performance for each feature setup. The best *MAE* result (1.398 × 10^−3^) was obtained using the interaction linear model when the minimum number of features was included. This result could be considered as adequate considering the small size of the dataset and the magnitude order of the response: defect length (0.01 ± 50% mmin Table 1).

However, the models that provided better predictive potentials were the stepwise and the interaction regression models. On the other hand, the performance results were not very suitable (maximum *R*2 is 0.65).

The decrease of the error when the “Contrast Rear” was excluded is shown in Table 4. In this case, when “Contrast Rear” ($\Delta T_{R}$) was not considered, the model performance increased in terms of *MAE* and *RMSE* (both are reduced) (Table 4). Moreover, in this case, the results of the deviation values for *MAE*, when different k-fold parameters were applied, can be higher in some cases (up to 26.86% increase for stepwise regression model).

Once 100 sets of values were studied, the experiment was repeated considering 500 sets of values (Table 5, Table 6, Table 7 and Table 8). In this case, the model typologies that provided the least amount of error were the GPR, specifically the square exponential (*MAE* = 6.665 × 10^−4^, *R*2 = 0.92) and the rational quadratic GPR (*MAE* = 6.666 × 10^−4^, *R*2 = 0.92), when the feature “Contrast Rear” ($\Delta T_{R}$) was considered and the defect features (thickness and emissivity coefficient) were excluded (Table 5 and Table 6). Note that the training time used when the rational quadratic kernel was chosen is three times higher than square exponential kernel, as is shown in the Appendix A. In this way, a model based on the rest of features using GPR provided a high performance.

However, training time was also higher in comparison with the other methods, especially the SVM (considering that four kernel functions and specifically three for RBF in function of kernel scale—fine, medium, and coarse—were considered), but these last ones provided a lower predictive model performance for the same setup (e.g., quadratic SVM provided *MAE* = 1.302 × 10^−3^ and *R*2 = 0.66). *RMSE* results calculated using SVM demonstrated that the outliers have an important effect (*RMSE* was significantly much higher than *MSA*). In addition, these regression models were shown for being the least sensitive to sample size because they were the only ones that at least provided acceptable results with 100 sets of values.

The interaction and stepwise regression models also provided adequate performance results, specifically the interaction regression, when all features were considered (*MAE* = 9.588 × 10^−4^, *R*2 = 0.81) (Table 5). The results showed a higher error than the GPR models, which is compatible with complex noisy non-linear functions [33]. Nevertheless, interaction regression required significantly less computational time (except for the stepwise regression model, which took very much longer). The difference between the different k-fold’s validations used was less than in the previous dataset for the same type of model, possibly due to the larger size of the dataset, as is shown in Appendix A.

**Figure 8:**
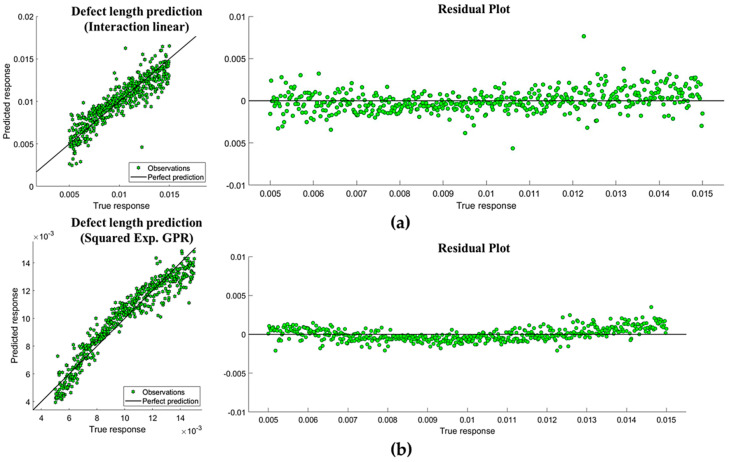
(**a**) Interaction regression model for all the features (*R*2 = 0.81, *MAE* = 9.588 × 10^−4^). (**b**) Gaussian Regression model for 8 features (*R*2 = 0.92, MAE = 6.666 × 10^−4^), including “Contrast Rear” and excluding defect thickness and emissivity coefficient.

Once we observed that both regression models and GPR models provided more adequate predictive results, a correlation between the observed and the prediction response was plotted. The two models of each type with lower error and the best fit are shown in Figure 8. Residuals were approximately and symmetrically distributed for the regression model (being a favorable aspect for the suitability of the model), as well as non-linearly distributed for the GPR.

Finally, MLP and RF models were the fastest training algorithms (Table 6, Table 7 and Table 8), but the *MAE* was significantly higher than the other models, indicating that they tend to improve for cases where fewer predictive features are used. Moreover, the rest of the performance parameters were less suitable than other models for the chosen configuration and setup.

When the “Contrast Rear” feature was excluded (Table 7 and Table 8), the highest performance model was the GPR (Table 7), especially for both square exponential (*R*2 = 0.86, *MAE* = 8.513 × 10^−4^) and rational quadratic (*R*2 = 0.86, *MAE* = 8.531 × 10^−4^) kernels. When *MAE* results were compared between the models, which included the “Constant Rear” feature ($\Delta T_{R}$), an increase in the *MAE* was detected for almost all trainline models (Table 9). In this way, there were models that “suffer less” from the loss of that feature: *MAE* increased when GPR models were used while the models where *MAE* increased less were SVM, RF, and MLP. The GPR models were more sensitive to the absence of such property than the regression models. In this manner, we can indicate, in general terms, that the “Contrast Rear” feature increased the predictive model performance, but this increase was not always significant (Table 9).

## 4. Conclusions

Using Python, a parametrical FEM model was prepared to study the effect that the presence of an internal defect generates on the temperature distribution of a thermally loaded solid. To check that the model worked correctly, a first battery of tests was carried out using the same geometries and materials employed by other authors [7,9,14]. Once it was checked that the thermal distribution results of these tests coincided with those from the authors in both shapes and values. The model was used to study how the thermal properties of the material $\left(c,k,\rho ,T_{E},\;\varepsilon ,\;h,T_{H}\right)$ and the geometric variables of the defect ($t_{D},L_{D}$) affected some interesting contrast values ($\Delta T_{F},\;\Delta T_{R}$), which were defined in Section 3.1. As a result, the influence of each geometric and thermal parameter (Table 1) over the contrast values were obtained (Figure 5).

In a second step, the simulation output frames were used as input to train 474 different prediction models to estimate the possibility of using thermal parameters $\left(c,k,\rho ,T_{E},\;\varepsilon ,\;h,T_{H},\;\Delta T_{F},\;\Delta T_{R}\right)$ and thus to predict the geometric features of the defect ($t_{D},L_{D}$). Different models in function of different features were established, trained, evaluated, and, finally, compared. The comparison of the different algorithms was the main contribution of this work.

Regarding defect thickness, it is possible to provide predictive models with moderate predictive performance. In particular, interaction linear regression and stepwise regression models provided adequate results. However, stepwise model was slower to train. The best model for defect thickness prediction using five features ($k,\;h,T_{H},\Delta T_{F},\;\Delta T_{R}$) was interaction linear regression (*MAE* = 7.038 × 10^−5^, *R*2 = 0.63). Using all the features the model gave a *MAE* of 5.148 × 10^−5^ (*R*2 = 0.79). In this case the “Contrast Rear” feature ($\Delta T_{R}$) was necessarily included in the model to get adequate results.

It was also possible to make predictive models for the defect length or thickness. In this case, a higher result for a higher number of model’s types was reported. When 100 sets of values were applied to train the models, only regression models provided adequate results, while if 500 sets of values were applied, different type of models gave adequate results. These models can be established both considering the “Contrast Rear” feature ($\Delta T_{R}$) and without considering it. However, when it is considered, the error tends to reduce (Table 10) and, consequently, the model performance improves despite the possible tendency towards collinearity between “Contrast Rear” and “Contrast Front” features. When “Contrast Rear” feature was considered, the best model was GPR based on a square exponential kernel that provided *MAE* of 6.665 × 10^−4^ when defect thickness and emissivity coefficient were also excluded.

Regression models were also tested and these gave adequate performance results but more unfavorable than those provided by GPR models (interaction regression model gave *MAE* of 9.588 × 10^−4^ and *R*2 of 0.81 when all features were used). *MAE* slightly increased when “Contrast Rear” feature was not considered (*MAE* = 1.183 × 10^−3^ and *R*2 = 0.74) and increased as the different variables were excluded (for the minimum numbers of features: *MAE* = 1.628 × 10^−3^ and *R*2 = 0.53). It was demonstrated that, for this case, the stepwise regression model did not provide significantly better results than the interaction regression models but significantly increased computational training time. However, the predicted versus actual plots showed an adequate linearity and constant variability for the interaction and stepwise regression models.

SVM were also models which allow the prediction of the defect length and their training times were very low, but their performances were less than the one obtained using GPR. However, a high outliers influence was detected for SVM model based on *RMSE* and *MAE* results, in predicted versus observed plots and was shown in the residual plots. If the weight given to the more extreme residuals is less, these models can be useful [31]. Additionally, MLP and RF methods provided predictions very quickly, but theirs performs were significantly worse than other indicated methods. A qualitative comparison based on the information obtained in this research is outlined in Table 10.

The key variables to establish an adequate predictive model for the different performed experiments were compatible with the weight given by the simulation results (Figure 5). The predictive performance was improved using both front and rear contrast data ($\Delta T_{F},\;\Delta T_{R}$). Monitoring of both sample’s sides improved predictive performance but, in the case of defect length prediction, adequate results could be also obtained from monitoring the front surface (reflection).

Futures lines will address the testing of the calculated algorithms from experimental results and a deeper study of the regression models modifying different parameters, especially in the case of the multilayer perceptron. Moreover, a mesostructural model should be proposed to take into account the presence of pores provoked by the material deposition process, which can be confused with small defects and cause variations in the mechanical properties in the different points and directions.

## Figures and Tables

**Table 1:** Material properties correspond to Nylon PA-12 [7]. Ranges of variation of the input variables are in absolute values and %.

Feature		Description	Initial Value	Lower Range (%)	Upper Range (%)
Defect thickness	$t_{D}$ (m)	Thickness of the defect	0.00050	0.00025 (−50%)	0.00075 (+50%)
Defect length	$L_{D}$ (m)	Length of the quadrangular side of the defect	0.010	0.005 (−50%)	0.015 (+50%)
Specific heat	c (J/kgK)	Capacity to absorb heat in the material	1590	795 (−50%)	2385 (+50%)
Conductivity coef.	k (W/m^2^ K)	Capacity to transfer heat inside the material	0.22	0.11 (−50%)	0.33 (+50%)
Density	ρ (kg/m^3)^)	Mass divided by volume in the material	1100	550 (−50%)	1650 (+50%)
Environment temperature	$T_{E}$ (°C)	Temperature of the air room during the experiment	24.4	12.2 (−50%)	36.6 (+50%)
Emissivity coef.	ε	For radiation heat transfer between the material and the environment	0.9500	0.9025 (−5%)	0.9975 (+5%)
Film coef.	h (W/m^2^/°C)	For convention heat transfer between the material and the environment	10.50	5.25 (−50%)	15.75 (+50%)
Max. heating temperature	$T_{H}$ (°C)	Maximum temperature applied to the upper surface during the heating step	120	60 (−50%)	180 (+50%)
Contrast front	$\Delta T_{F}$ (°C)	Maximum difference between *P1* and *P2* temperatures	Output of the FEM simulation		
Contrast rear	$\Delta T_{R}$ (°C)	Maximum difference between *P4* and *P3* temperatures	Output of the FEM simulation		

**Table 2:** Performance results for defect thickness predictor models using a dataset of 500 sets of values. The models with the best predictive performance are indicated in bold type.

	500 Data	Regression		
		Linear	Interaction	Stepwise
None	*RMSE*	1.046 × 10^−4^	**6.560 × 10^−5^**	8.234 × 10^−5^
	*R*2	0.47	**0.79**	0.68
	*MAE*	8.690 × 10^−5^	**5.148 × 10^−5^**	6.612 × 10^−5^
$L_{D}$	*RMSE*	1.056 × 10^−4^	**7.388 × 10^−5^**	8.153 × 10^−5^
	*R*2	0.47	**0.74**	0.68
	*MAE*	8.720 × 10^−5^	**5.884 × 10^−5^**	6.492 × 10^−5^
$L_{D},\;\varepsilon$	*RMSE*	1.070 × 10^−4^	**7.286 × 10^−5^**	8.320 × 10^−5^
	*R*2	0.46	**0.75**	0.67
	*MAE*	8.710 × 10^−5^	**5.777 × 10^−5^**	6.610 × 10^−5^
$L_{D},\;\varepsilon ,\;T_{E}$	*RMSE*	1.053 × 10^−4^	**7.575 × 10^−5^**	8.353 × 10^−5^
	*R*2	0.47	**0.73**	0.67
	*MAE*	8.688 × 10^−5^	**5.985 × 10^−5^**	6.655 × 10^−5^
$L_{D},\;\varepsilon ,\;T_{E}$, c,	*RMSE*	1.060 × 10^−4^	**8.290 × 10^−5^**	8.562 × 10^−5^
	*R*2	0.46	**0.67**	0.65
	*MAE*	8.730 × 10^−5^	**6.675 × 10^−5^**	6.890 × 10^−5^
$L_{D},\;\varepsilon ,\;T_{E}$, c, ρ	*RMSE*	1.080 × 10^−4^	**8.773 × 10^−5^**	8.886 × 10^−5^
	*R*2	0.45	**0.63**	0.62
	*MAE*	8.910 × 10^−5^	**7.038 × 10^−5^**	7.134 × 10^−5^

**Table 3:** Performance results for defect length predictor models using a dataset of 100 sets of values. The models with the best predictive performance are indicated in bold type.

		Considering Contrast Rear			Without Contrast Rear		
		Regression			Regression		
Excluding Features		Linear	Interaction	Stepwise	Linear	Interaction	Stepwise
None	*RMSE*	2.671 × 10^−3^	3.330 × 10^−3^	**1.949 × 10^−3^**	2.440 × 10^−3^	1.995 × 10^−3^	**1.853 × 10^−3^**
	*R*2	0.19	0.25	**0.57**	0.32	0.54	**0.61**
	*MAE*	2.180 × 10^−3^	2.299 × 10^−3^	**1.506 × 10^−3^**	2.027 × 10^−3^	1.534 × 10^−3^	**1.470 × 10^−3^**
$t_{D}$	*RMSE*	2.633 × 10^−3^	2.382 × 10^−3^	**1.864 × 10^−3^**	2.423 × 10^−3^	2.008 × 10^−3^	**1.736 × 10^−3^**
	*R*2	0.22	0.36	**0.61**	0.32	0.54	**0.65**
	*MAE*	2.164 × 10^−3^	1.719 × 10^−3^	**1.439 × 10^−3^**	2.033 × 10^−3^	1.624 × 10^−3^	**1.400 × 10^−3^**
$t_{D},\;\varepsilon$	*RMSE*	2.613 × 10^−3^	2.144 × 10^−3^	**1.854 × 10^−3^**	2.405 × 10^−3^	1.821 × 10^−3^	**1.782 × 10^−3^**
	*R*2	0.23	0.48	**0.61**	0.33	0.62	**0.63**
	*MAE*	2.156 × 10^−3^	1.527 × 10^−3^	**1.456 × 10^−3^**	2.015 × 10^−3^	1.507 × 10^−3^	**1.509 × 10^−3^**
$t_{D},\;\varepsilon ,\;T_{E}$	*RMSE*	2.556 × 10^−3^	2.040 × 10^−3^	**1.770 × 10^−3^**	2.392 × 10^−3^	1.859 × 10^−3^	**1.804 × 10^−3^**
	*R*2	0.26	0.53	**0.65**	0.34	0.6	**0.63**
	*MAE*	2.108 × 10^−3^	**1.482 × 10^−3^**	**1.411 × 10^−3^**	1.994 × 10^−3^	**1.443 × 10^−3^**	1.409 × 10^−3^
$t_{D},\;\varepsilon ,\;T_{E}$,*k*	*RMSE*	2.494 × 10^−3^	**1.820 × 10^−3^**	1.893 × 10^−3^	2.452 × 10^−3^	**2.092 × 10^−3^**	2.132 × 10^−3^
	*R*2	0.3	**0.63**	0.6	0.31	**0.5**	0.48
	*MAE*	2.013 × 10^−3^	**1.398 × 10^−3^**	1.471 × 10^−3^	2.021 × 10^−3^	**1.689 × 10^−3^**	1.764 × 10^−3^

**Table 4:** Variation of *MAE* (100 sets of values) when “Contrast Rear” is excluded as predictive feature calculated as:$\frac{MAE_{without\;\Delta T_{R}}-MAE_{with\;\Delta T_{R}}}{MAE_{with\;\Delta T_{R}}}\times 100$.

Excluding Features:	Linear	Interaction	Stepwise
None	−8.65%	−40.09%	−4.94%
	−7.03%	−33.28%	−2.42%
$t_{D}$	−7.96%	−15.69%	−6.86%
	−6.07%	−5.50%	−2.71%
$t_{D},\;\varepsilon$	−7.98%	−15.07%	−3.87%
	−6.52%	−1.32%	3.65%
$t_{D},\;\varepsilon ,\;T_{E}$	−6.45%	−8.85%	1.93%
	−5.40%	−2.64%	−0.11%
$t_{D},\;\varepsilon ,\;T_{E},\;k$	−1.70%	14.96%	12.65%
	0.43%	20.78%	19.93%

**Table 5:** Performance results for defect length predictor models using a dataset of 500 sets of values. Part 1: Regression and Gaussian process regression model (GPR) when “Contrast Rear” is contemplated as feature. The models with the best predictive performance are indicated in bold type.

		Regression			Gaussian Processes Regression		
Excluding Features		Linear	Interaction	Stepwise	Square ExpGPR	Matern 5/2GPR	Rational Quadratic GPR
None	*RMSE*	2.273 × 10^−3^	1.276 × 10^−3^	1.278 × 10^−3^	**9.247 × 10^−4^**	9.460 × 10^−4^	9.252 × 10^−4^
	*R*2	0.38	0.81	0.81	**0.90**	0.89	0.90
	*MAE*	1.805 × 10^−3^	9.588 × 10^−4^	9.594 × 10^−4^	**7.256 × 10^−4^**	7.505 × 10^−4^	7.267 × 10^−4^
$t_{D}$	*RMSE*	2.292 × 10^−3^	1.367 × 10^−3^	1.489 × 10^−3^	9.963 × 10^−4^	**8.720 × 10^−4^**	9.975 × 10^−4^
	*R*2	0.37	0.78	0.74	0.88	**0.91**	0.88
	*MAE*	1.800 × 10^−3^	9.663 × 10^−4^	1.017 × 10^−3^	6.967 × 10^−4^	**6.940 × 10^−4^**	6.976 × 10^−4^
$t_{D},\;\varepsilon$	*RMSE*	2.259 × 10^−3^	1.341 × 10^−3^	1.32100B7×10^−3^	**8.221 × 10^−4^**	8.546 × 10^−4^	8.221 × 10^−4^
	*R*2	0.39	0.79	0.79	**0.92**	0.91	0.92
	*MAE*	1.804 × 10^−3^	1.027 × 10^−3^	1.031 × 10^−3^	**6.665 × 10^−^^4^**	6.967 × 10^−4^	6.666 × 10^−4^
$t_{D},\;\varepsilon ,\;T_{E}$	*RMSE*	2.277 × 10^−3^	1.485 × 10^−3^	1.478 × 10^−3^	**1.172 × 10^−3^**	1.173 × 10^−3^	1.172 × 10^−3^
	*R*2	0.38	0.74	0.74	**0.84**	0.84	0.84
	*MAE*	1.819 × 10^−3^	1.156 × 10^−3^	1.152 × 10^−3^	**9.310 × 10^−4^**	9.374 × 10^−4^	9.310 × 10^−4^
$t_{D},\;\varepsilon ,\;T_{E},\;k$	*RMSE*	2.264 × 10^−3^	1.447 × 10^−3^	1.454 × 10^−3^	**1.158 × 10^−3^**	1.153 × 10^−3^	1.158 × 10^−3^
	*R*2	0.39	0.75	0.75	**0.84**	0.84	0.84
	*MAE*	1.810 × 10^−3^	1.130 × 10^−3^	1.144 × 10^−3^	**9.120 × 10^−4^**	9.170 × 10^−4^	9.120 × 10^−4^

**Table 6:** Performance results for defect length predictor models using a dataset of 500 sets of values. Part 2: support vector machines (SVM), multilayer perceptron (MLP), and random forest (RF) when “Contrast Rear” is contemplated as feature.

		SVM			Multilayer Perceptron	Random Forest
Excluding Features:		Cubic	Quadratic	Medium Gaussian		
None	*RMSE*	1.774 × 10^−3^	1.622 × 10^−3^	1.756 × 10^−3^	2.241 × 10^−3^	2.376 × 10^−3^
	*R*2	0.63	0.69	0.63	0.55	0.36
	*MAE*	1.180 × 10^−3^	1.246 × 10^−3^	1.423 × 10^−3^	1.741 × 10^−3^	2.031 × 10^−3^
$t_{D}$	*RMSE*	2.784 × 10^−3^	1.713 × 10^−3^	1.737 × 10^−3^	2.080 × 10^−3^	2.340 × 10^−3^
	*R*2	0.08	0.65	0.64	0.60	0.38
	*MAE*	1.210 × 10^−3^	1.298 × 10^−3^	1.383 × 10^−3^	1.650 × 10^−3^	1.990 × 10^−3^
$t_{D},\;\varepsilon$	*RMSE*	1.961 × 10^−3^	1.684 × 10^−3^	1.744 × 10^−3^	2.070 × 10^−3^	2.280 × 10^−3^
	*R*2	0.54	0.66	0.64	0.63	0.42
	*MAE*	1.204 × 10^−3^	1.302 × 10^−3^	1.373 × 10^−3^	1.640 × 10^−3^	1.940 × 10^−3^
$t_{D},\;\varepsilon ,\;T_{E}$	*RMSE*	1.900 × 10^−3^	1.708 × 10^−3^	1.731 × 10^−3^	2.060 × 10^−3^	2.270 × 10^−3^
	*R*2	0.57	0.65	0.64	0.60	0.42
	*MAE*	1.338 × 10^−3^	1.338 × 10^−3^	1.360 × 10^−3^	1.640 × 10^−3^	1.930 × 10^−3^
$t_{D},\;\varepsilon ,\;T_{E},\;k$	*RMSE*	1.708 × 10^−3^	1.747 × 10^−3^	1.720 × 10^−3^	2.130 × 10^−3^	2.200 × 10^−3^
	*R*2	0.65	0.64	0.64	0.58	0.46
	*MAE*	1.284 × 10^−3^	1.358 × 10^−3^	1.379 × 10^−3^	1.690 × 10^−3^	1.870 × 10^−3^

**Table 7:** Performance results for defect length predictor models using a dataset of 500 sets of values. Part 1: Regression and GPR when “Contrast Rear” is not contemplated as feature. The models with the best predictive performance are indicated in bold type.

		Regression			Gaussian Processes Regression		
Excluding Features:		Linear	Interaction	Stepwise	Square ExpGPR	Matern 5/2GPR	Rational Quadratic GPR
None	*RMSE*	2.231 × 10^−3^	1.485 × 10^−3^	1.466 × 10^−3^	1.072 × 10^−3^	1.103 × 10^−3^	**1.073 × 10^−3^**
	*R*2	0.41	0.74	0.75	0.86	0.86	**0.86**
	*MAE*	1.793 × 10^−3^	1.183 × 10^−3^	1.168 × 10^−3^	8.513 × 10^−4^	8.826 × 10^−4^	**8.531 × 10^−4^**
$t_{D}$	*RMSE*	2.285 × 10^−3^	1.727 × 10^−3^	1.724 × 10^−3^	**1.501 × 10^−3^**	1.505 × 10^−3^	1.499 × 10^−3^
	*R*2	0.38	0.65	0.65	**0.73**	0.73	0.73
	*MAE*	1.863 × 10^−3^	1.383 × 10^−3^	1.379 × 10^−3^	**1.208 × 10^−3^**	1.215 × 10^−3^	1.209 × 10^−3^
$t_{D},\;\varepsilon$	*RMSE*	2.277 × 10^−3^	1.485 × 10^−3^	1.478 × 10^−3^	**1.172 × 10^−3^**	1.173 × 10^−3^	1.172 × 10^−3^
	*R*2	0.38	0.74	0.74	**0.84**	0.84	0.84
	*MAE*	1.819 × 10^−3^	1.156 × 10^−3^	1.152 × 10^−3^	**9.310 × 10^−4^**	9.374 × 10^−4^	9.310 × 10^−4^
$t_{D},\;\varepsilon ,\;T_{E}$	*RMSE*	2.301 × 10^−3^	1.809 × 10^−3^	1.837 × 10^−3^	**1.624 × 10^−3^**	1.631 × 10^−3^	1.625 × 10^−3^
	*R*2	0.37	0.61	0.6	**0.69**	0.68	0.69
	*MAE*	1.881 × 10^−3^	1.453 × 10^−3^	1.473 × 10^−3^	**1.301 × 10^−3^**	1.309 × 10^−3^	1.302 × 10^−3^
$t_{D},\;\varepsilon ,\;T_{E},\;k$	*RMSE*	2.323 × 10^−3^	1.984 × 10^−3^	1.986 × 10^−3^	**1.864 × 10^−3^**	1.866 × 10^−3^	1.866 × 10^−3^
	*R*2	0.36	0.53	0.53	**0.59**	0.59	0.59
	*MAE*	1.913 × 10^−3^	1.628 × 10^−3^	1.631 × 10^−3^	**1.516 × 10^−3^**	1.517 × 10^−3^	1.517 × 10^−3^

**Table 8:** Performance results for defect length predictor models using a dataset of 500 sets of values. Part 2: SVM, MLP, and RF when “Contrast Rear” is not contemplated as feature.

		SVM			Multilayer Perceptron	Random Forest
		Cubic	Quadratic	Medium Gaussian		
None	*RMSE*	1.677 × 10^−3^	1.829 × 10^−3^	2.000 × 10^−3^	2.290 × 10^−3^	2.490 × 10^−3^
	*R*2	0.67	0.60	0.53	0.54	0.30
	*MAE*	1.276 × 10^−3^	1.440 × 10^−3^	1.614 × 10^−3^	1.810 × 10^−3^	2.140 × 10^−3^
$t_{D}$	*RMSE*	1.845 × 10^−3^	1.944 × 10^−3^	2.066 × 10^−3^	2.350 × 10^−3^	2.480 × 10^−3^
	*R*2	0.60	0.55	0.49	0.52	0.29
	*MAE*	1.443 × 10^−3^	1.540 × 10^−3^	1.662 × 10^−3^	1.890 × 10^−3^	2.120 × 10^−3^
$t_{D},\;\varepsilon$	*RMSE*	1.900 × 10^−3^	1.708 × 10^−3^	1.731 × 10^−3^	2.060 × 10^−3^	2.270 × 10^−3^
	*R*2	0.57	0.65	0.64	0.59751354	0.421201
	*MAE*	1.338 × 10^−3^	1.338 × 10^−3^	1.360 × 10^−3^	1.640 × 10^−3^	1.930 × 10^−3^
$t_{D},\;\varepsilon ,\;T_{E}$	*RMSE*	1.991 × 10^−3^	2.029 × 10^−3^	2.073 × 10^−3^	2.400 × 10^−3^	2.400 × 10^−3^
	*R*2	0.53	0.51	0.49	0.5	0.34
	*MAE*	1.557 × 10^−3^	1.606 × 10^−3^	1.650 × 10^−3^	1.930 × 10^−3^	2.050 × 10^−3^
$t_{D},\;\varepsilon ,\;T_{E},\;k$	*RMSE*	2.296 × 10^−3^	2.333 × 10^−3^	2.151 × 10^−3^	2.520 × 10^−3^	2.340 × 10^−3^
	*R*2	0.37	0.35	0.45	0.45	0.37
	*MAE*	1.793 × 10^−3^	1.834 × 10^−3^	1.722 × 10^−3^	2.040 × 10^−3^	1.980 × 10^−3^

**Table 9:** Variation of *MAE* (500 sets of values) when “Contrast Rear” is excluded as predictive feature calculated as: $\frac{MAE_{without\;\Delta T_{R}}-MAE_{with\;\Delta T_{R}}}{MAE_{with\;\Delta T_{R}}}\times 100$.

		Regression			Gaussian Regression Model			SVM			Multilayer Perceptron	Random Forest
Excluding Feature:		Linear	Interaction	Stepwise	Square ExpGPR	Matern 5/2GPR	Rational Quadratic	Cubic	Quadratic	Medium Gaussian		
None	*RMSE*	−1.85%	16.30%	14.77%	15.93%	16.64%	15.97%	−5.45%	12.74%	13.87%	2.19%	4.80%
	*MAE*	−0.68%	23.33%	21.76%	17.33%	17.60%	17.39%	8.13%	15.57%	13.41%	3.96%	5.37%
$t_{D}$	*RMSE*	−0.34%	26.31%	15.73%	50.66%	72.61%	50.25%	−33.73%	13.44%	18.95%	12.98%	5.98%
	*MAE*	3.51%	43.12%	35.64%	73.39%	75.01%	73.34%	19.21%	18.59%	20.16%	14.55%	6.53%
$t_{D},\;\varepsilon$	*RMSE*	0.79%	10.72%	11.89%	42.53%	37.30%	42.52%	−3.10%	1.44%	−0.74%	−0.48%	−0.44%
	*MAE*	0.81%	12.51%	11.74%	39.66%	34.56%	39.65%	11.13%	2.78%	−0.92%	0.00%	−0.52%
$t_{D},\;\varepsilon ,\;T_{E}$	*RMSE*	1.03%	21.86%	24.22%	38.61%	39.00%	38.70%	4.79%	18.79%	19.76%	16.50%	5.73%
	*MAE*	3.41%	25.70%	27.84%	39.71%	39.62%	39.87%	16.33%	19.99%	21.30%	17.68%	6.22%
$t_{D},\;\varepsilon ,\;T_{E},\;k$	*RMSE*	2.61%	37.08%	36.58%	61.02%	61.86%	61.20%	34.44%	33.54%	25.03%	18.31%	6.36%
	*MAE*	5.66%	44.03%	42.56%	66.18%	65.39%	66.35%	39.64%	35.06%	24.84%	20.71%	5.88%
	Mean %	1.50%	26.10%	24.27%	44.50%	45.96%	44.52%	9.14%	17.19%	15.57%	10.64%	4.59%

**Table 10:** Qualitative comparison of the predictive models based on results.

		Prediction Model for Defect Length					Prediction Model for Defect Thickness		
Model		Performance	Processing Time	Outliers Influence	Sensitive to Lack of Contrast Front	Sample Size Sensitive *	Performance	Processing Time	Outliers Sensitive
**Regression**	**Linear**	Very low	Low	High	Low	Moderate	Low	Low	Low
	**Interaction**	High	Low	Moderate	High	Moderate	Moderate	Low	Low
	**Stepwise**	High	Very high	Low	High	Moderate	Moderate	Very high	Low
**GPR**	**Square exp.**	Very high	Moderate	Low	Very high	High			
	**Matern 5/2**	Very high	Moderate	Low	Very high	High			
	**Rational Quadratic**	Very high	High	Low	Very high	High			
**SVM**	**Cubic**	Moderate	Very low	High	Low	High			
	**Quadratic**	Moderate	Very low	Medium	Moderate	High			
	**Gaussian Medium**	Moderate	Very low	Medium	Moderate	High			
**Multilayer Perceptron**		Low	Very low	Moderate	Low	High			
**Random Forest**		Very low	Very low	Low	Very low	High			

* Only based on the experiments with two datasets (100 and 500 sets of values).

## Appendix A

All the parameters analyzed for the different predictive models with the different configurations are outlined in this section (Table A1, Table A2, Table A3, Table A4, Table A5 and Table A6). Please note that only *MAE*, *R2,* and *RMSE* are reported in the manuscript but the rest of the parameters for each trained model is given here to facilitate its reading.

**Table A1:** Performance results for thickness predictor models using dataset of 500 sets of values.

Excluding Features	500 Data	Regression		
		Linear	Interaction	Stepwise
**None**	*RMSE*	1.046 × 10^−4^	6.560 × 10^−5^	8.234 × 10^−5^
	*R*2	0.47	0.79	0.68
	*MAE*	8.690 × 10^−5^	5.148 × 10^−5^	6.612 × 10^−5^
	Training time	0.499	0.610	108.630
	Dev.MAE 5 Folder	−0.09%	0.21%	1.63%
	Dev.MAE 15 Folder	−0.60%	−0.04%	2.23%
$L_{D}$	*RMSE*	1.056 × 10^−4^	7.388 × 10^−5^	8.153 × 10^−5^
	*R*2	0.47	0.74	0.68
	*MAE*	8.720 × 10^−5^	5.884 × 10^−5^	6.492 × 10^−5^
	Training time	0.454	0.494	54.771
	Dev.MAE 5 Folder	−0.09%	2.20%	2.06%
	Dev.MAE 15 Folder	−1.44%	−1.39%	−2.45%
$L_{D},\;\varepsilon$	*RMSE*	1.070 × 10^−4^	7.286 × 10^−5^	8.320 × 10^−5^
	*R*2	0.46	0.75	0.67
	*MAE*	8.710 × 10^−5^	5.777 × 10^−5^	6.610 × 10^−5^
	Training time	0.430	0.432	33.783
	Dev.MAE 5 Folder	−0.19%	2.09%	1.91%
	Dev.MAE 15 Folder	0.46%	−1.58%	0.37%
$L_{D},\;\varepsilon ,\;T_{E}$	*RMSE*	1.053 × 10^−4^	7.575 × 10^−5^	8.353 × 10^−5^
	*R*2	0.47	0.73	0.67
	*MAE*	8.688 × 10^−5^	5.985 × 10^−5^	6.655 × 10^−5^
	Training time	0.445	0.397	22.331
	Dev.MAE 5 Folder	−0.51%	−1.76%	2.55%
	Dev.MAE 15 Folder	−0.09%	1.26%	−0.80%
$L_{D},\;\varepsilon ,\;T_{E}$, c,	*RMSE*	1.060 × 10^−4^	8.290 × 10^−5^	8.562 × 10^−5^
	*R*2	0.46	0.67	0.65
	*MAE*	8.730 × 10^−5^	6.675 × 10^−5^	6.890 × 10^−5^
	Training time	0.443	0.394	11.998
	Dev.MAE 5 Folder	0.59%	−0.87%	−0.60%
	Dev.MAE 15 Folder	−0.37%	1.87%	2.01%
$L_{D},\;\varepsilon ,\;T_{E}$, c, ρ	*RMSE*	1.080 × 10^−4^	8.773 × 10^−5^	8.886 × 10^−5^
	*R*2	0.45	0.63	0.62
	*MAE*	8.910 × 10^−5^	7.038 × 10^−5^	7.134 × 10^−5^
	Training time	0.490	0.427	6.739
	Dev.MAE 5 Folder	−0.29%	−3.04%	0.56%
	Dev.MAE 15 Folder	0.38%	−0.85%	−1.71%

**Table A2:** Performance results for defect length predictor models using dataset of 100 sets of values.

		Considering Contrast Rear			Without Contrast Rear		
		Regression			Regression		
Excluding Features		Linear	Interaction	Stepwise	Linear	Interaction	Stepwise
None	*RMSE*	2.671 × 10^−3^	3.330 × 10^−3^	1.949 × 10^−3^	2.440 × 10^−3^	1.995 × 10^−3^	1.853 × 10^−3^
	*R*2	0.19	0.25	0.57	0.32	0.54	0.61
	*MAE*	2.180 × 10^−3^	2.299 × 10^−3^	1.506 × 10^−3^	2.027 × 10^−3^	1.534 × 10^−3^	1.470 × 10^−3^
	Training time	0.321	0.303	32.787	0.416	0.477	30.602
	Dev.MAE 5 Folder	−8.49%	−16.74%	20.36%	−2.86%	10.10%	−3.21%
	Dev.MAE 15 Folder	−4.60%	−27.24%	−5.04%	−1.14%	10.58%	−6.36%
$t_{D}$	*RMSE*	2.633 × 10^−3^	2.382 × 10^−3^	1.864 × 10^−3^	2.423 × 10^−3^	2.008 × 10^−3^	1.736 × 10^−3^
	*R*2	0.22	0.36	0.61	0.32	0.54	0.65
	*MAE*	2.164 × 10^−3^	1.719 × 10^−3^	1.439 × 10^−3^	2.033 × 10^−3^	1.624 × 10^−3^	1.400 × 10^−3^
	Training time	0.304	0.286	20.397	0.518	0.618	29.631
	Dev.MAE 5 Folder	−8.50%	−13.81%	−3.51%	0.22%	6.47%	2.94%
	Dev.MAE 15 Folder	−4.87%	−15.70%	−3.88%	−8.06%	−15.87%	−1.49%
$t_{D},\;\varepsilon$	*RMSE*	2.613 × 10^−3^	2.144 × 10^−3^	1.854 × 10^−3^	2.405 × 10^−3^	1.821 × 10^−3^	1.782 × 10^−3^
	*R*2	0.23	0.48	0.61	0.33	0.62	0.63
	*MAE*	2.156 × 10^−3^	1.527 × 10^−3^	1.456 × 10^−3^	2.015 × 10^−3^	1.507 × 10^−3^	1.509 × 10^−3^
	Training time	0.320	0.261	14.640	0.536	0.548	20.132
	Dev.MAE 5 Folder	−9.18%	−17.08%	−10.89%	−0.04%	3.40%	−5.42%
	Dev.MAE 15 Folder	−6.16%	−14.08%	−4.83%	−7.52%	−10.13%	−8.80%
$t_{D},\;\varepsilon ,\;T_{E}$	*RMSE*	2.556 × 10^−3^	2.040 × 10^−3^	1.770 × 10^−3^	2.392 × 10^−3^	1.859 × 10^−3^	1.804 × 10^−3^
	*R*2	0.26	0.53	0.65	0.34	0.6	0.63
	*MAE*	2.108 × 10^−3^	1.482 × 10^−3^	1.411 × 10^−3^	1.994100^−3^	1.443 × 10^−3^	1.409 × 10^−3^
	Training time	0.332	0.249	10.105	0.528	0.509	13.732
	Dev.MAE 5 Folder	−6.55%	−5.71%	−1.92%	1.51%	7.46%	26.86%
	Dev.MAE 15 Folder	−4.30%	0.76%	5.47%	−5.57%	−0.15%	3.52%
$t_{D},\;\varepsilon ,\;T_{E}$, *k*	*RMSE*	2.494 × 10^−3^	1.820 × 10^−3^	1.893 × 10^−3^	2.452 × 10^−3^	2.092 × 10^−3^	2.132 × 10^−3^
	*R*2	0.3	0.63	0.6	0.31	0.5	0.48
	*MAE*	2.013 × 10^−3^	1.398 × 10^−3^	1.471 × 10^−3^	2.021 × 10^−3^	1.689 × 10^−3^	1.764 × 10^−3^
	Training time	0.356	0.269	7.293	0.482	0.396	5.240
	Dev.MAE 5 Folder	−2.69%	−7.08%	4.25%	−3.41%	−15.14%	−15.26%
	Dev.MAE 15 Folder	0.17%	5.97%	12.83%	−5.51%	−3.95%	−7.81%

**Table A3:** Performance results for defect length predictor models using dataset of 500 sets of values. Regression and GPR when “Contrast Rear” is contemplated as feature.

		Regression			Gaussian Processes Regression		
Excluding Features		Linear	Interaction	Stepwise	Square ExpGPR	Matern 5/2GPR	Rational Quadratic GPR
None	*RMSE*	2.273 × 10^−3^	1.276 × 10^−3^	1.278 × 10^−3^	9.247 × 10^−4^	9.460 × 10^−4^	9.252 × 10^−4^
	*R*2	0.38	0.81	0.81	0.90	0.89	0.90
	*MAE*	1.805 × 10^−3^	9.588 × 10^−4^	9.594 × 10^−4^	7.256 × 10^−4^	7.505 × 10^−4^	7.267 × 10^−4^
	Training time	0.495	0.575	102.010	5.711	6.021	16.218
	Dev.MAE 5 Folder	−0.37%	2.98%	3.29%	3.78%	4.33%	3.69%
	Dev.MAE 15 Folder	−0.15%	1.48%	0.15%	−0.50%	0.02%	−0.56%
$t_{D}$	*RMSE*	2.292 × 10^−3^	1.367 × 10^−3^	1.489 × 10^−3^	9.963 × 10^−4^	8.720 × 10^−4^	9.975 × 10^−4^
	*R*2	0.37	0.78	0.74	0.88	0.91	0.88
	*MAE*	1.800 × 10^−3^	9.663 × 10^−4^	1.017 × 10^−3^	6.967 × 10^−4^	6.940 × 10^−4^	6.976 × 10^−4^
	Training time	0.501	0.562	65.640	6.768	8.278	17.785
	Dev.MAE 5 Folder	0.22%	8.04%	0.90%	8.08%	9.40%	7.98%
	Dev.MAE 15 Folder	0.82%	6.16%	−0.46%	2.96%	6.27%	2.97%
$t_{D},\;\varepsilon$	*RMSE*	2.259 × 10^−3^	1.341 × 10^−3^	1.321 × 10^−3^	8.221 × 10^−4^	8.546 × 10^−4^	8.221 × 10^−4^
	*R*2	0.39	0.79	0.79	0.92	0.91	0.92
	*MAE*	1.804 × 10^−3^	1.027 × 10^−3^	1.031 × 10^−3^	6.665 × 10^−4^	6.967 × 10^−4^	6.666 × 10^−4^
	Training time	0.628	0.714	47.615	5.339	3.418	15.990
	Dev.MAE 5 Folder	0.42%	4.66%	5.61%	3.65%	3.93%	3.67%
	Dev.MAE 15 Folder	−0.22%	−0.44%	−1.52%	−2.56%	−2.02%	−2.56%
$t_{D},\;\varepsilon ,\;T_{E}$	*RMSE*	2.277 × 10^−3^	1.485 × 10^−3^	1.478 × 10^−3^	1.172 × 10^−3^	1.173 × 10^−3^	1.172 × 10^−3^
	*R*2	0.38	0.74	0.74	0.84	0.84	0.84
	*MAE*	1.819 × 10^−3^	1.156 × 10^−3^	1.152 × 10^−3^	9.310 × 10^−4^	9.374 × 10^−4^	9.310 × 10^−4^
	Training time	0.561	0.477	26.148	3.549	4.602	12.395
	Dev.MAE 5 Folder	−0.42%	−0.22%	−0.35%	−1.00%	−0.84%	−0.99%
	Dev.MAE 15 Folder	0.20%	0.55%	0.71%	−1.11%	−1.17%	−1.11%
$t_{D},\;\varepsilon ,\;T_{E},\;k$	*RMSE*	2.264 × 10^−3^	1.447 × 10^−3^	1.454 × 10^−3^	1.158 × 10^−3^	1.153 × 10^−3^	1.158 × 10^−3^
	*R*2	0.39	0.75	0.75	0.84	0.84	0.84
	*MAE*	1.810 × 10^−3^	1.130 × 10^−3^	1.144 × 10^−3^	9.120 × 10^−4^	9.170 × 10^−4^	9.120 × 10^−4^
	Training time	0.635	0.532	16.650	5.453	6.153	15.873
	Desv MAE	0.72%	1.58%	1.66%	1.32%	5.02%	1.32%
	Desv MAE	0.50%	0.28%	0.05%	−0.54%	−0.65%	−0.55%

**Table A4:** Performance results for defect length predictor models using dataset of 500 sets of values. SVM, MLP, and RF when “Contrast Rear” is contemplated as feature.

		SVM			Multilayer Perceptron	Random Forest
Excluding Features		Cubic	Quadratic	Medium Gaussian		
None	*RMSE*	1.774 × 10^−3^	1.622 × 10^−3^	1.756 × 10^−3^	2.241 × 10^−3^	2.376 × 10^−3^
	*R*2	0.63	0.69	0.63	0.55	0.36
	*MAE*	1.180 × 10^−3^	1.246 × 10^−3^	1.423 × 10^−3^	1.741 × 10^−3^	2.031 × 10^−3^
	Training time	0.220	0.225	0.218	0.177	0.116
	Dev.MAE 5 Folder	7.22%	14.77%	6.28%	3.96%	1.67%
	Dev.MAE 15 Folder	−3.83%	1.07%	−0.72%	−1.78%	−0.10%
$t_{D}$	RMSE	2.784 × 10^−3^	1.713 × 10^−3^	1.737 × 10^−3^	2.080 × 10^−3^	2.340 × 10^−3^
	R2	0.08	0.65	0.64	0.60	0.38
	MAE	1.210 × 10^−3^	1.298 × 10^−3^	1.383 × 10^−3^	1.650 × 10^−3^	1.990 × 10^−3^
	Training time	0.255	0.259	0.222	0.165	0.107
	Dev.MAE 5 Folder	0.68%	7.22%	8.79%	3.03%	2.51%
	Dev.MAE 15 Folder	−0.60%	−5.78%	3.58%	−1.21%	−0.50%
$t_{D},\;\varepsilon$	*RMSE*	1.961 × 10^−3^	1.684 × 10^−3^	1.744 × 10^−3^	2.070 × 10^−3^	2.280 × 10^−3^
	*R*2	0.54	0.66	0.64	0.63	0.42
	*MAE*	1.204 × 10^−3^	1.302 × 10^−3^	1.373 × 10^−3^	1.640 × 10^−3^	1.940 × 10^−3^
	Training time	0.340	0.281	0.276	0.181	0.105
	Dev.MAE 5 Folder	1.15%	5.61%	2.78%	3.05%	2.06%
	Dev.MAE 15 Folder	−6.76%	−6.60%	1.26%	−3.66%	−0.52%
$t_{D},\;\varepsilon ,\;T_{E}$	*RMSE*	1.900 × 10^−3^	1.708 × 10^−3^	1.731 × 10^−3^	2.060 × 10^−3^	2.270 × 10^−3^
	*R*2	0.57	0.65	0.64	0.60	0.42
	*MAE*	1.338 × 10^−3^	1.338 × 10^−3^	1.360 × 10^−3^	1.640 × 10^−3^	1.930 × 10^−3^
	Training time	0.297	0.278	0.280	0.118	0.085
	Dev.MAE 5 Folder	1.27%	5.04%	12.06%	6.10%	1.55%
	Dev.MAE 15 Folder	−6.52%	4.62%	6.00%	2.44%	−1.04%
$t_{D},\;\varepsilon ,\;T_{E},\;k$	*RMSE*	1.708 × 10^−3^	1.747 × 10^−3^	1.720 × 10^−3^	2.130 × 10^−3^	2.200 × 10^−3^
	*R*2	0.65	0.64	0.64	0.58	0.46
	*MAE*	1.284 × 10^−3^	1.358 × 10^−3^	1.379 × 10^−3^	1.690 × 10^−3^	1.870 × 10^−3^
	Training time	0.419	0.436	0.326	0.088	0.085
	Desv MAE	15.26%	10.90%	7.29%	3.55%	1.60%
	Desv MAE	−0.31%	−0.37%	−0.43%	2.37%	−0.53%

**Table A5:** Performance results for defect length predictor models using dataset of 500 sets of values. Regression and GPR when “Contrast Rear” is not contemplated as feature.

		Regression			Gaussian Processes Regression		
Excluding Features		Linear	Interaction	Stepwise	Square ExpGPR	Matern 5/2GPR	Rational Quadratic GPR
None	*RMSE*	2.231 × 10^−3^	1.485 × 10^−3^	1.466 × 10^−3^	1.072 × 10^−3^	1.103 × 10^−3^	1.073 × 10^−3^
	*R*2	0.41	0.74	0.75	0.86	0.86	0.86
	*MAE*	1.793 × 10^−03^	1.183 × 10^−3^	1.168 × 10^−3^	8.513 × 10^−4^	8.826 × 10^−4^	8.531 × 10^−4^
	Training time	0.416	0.962	66.550	7.732	7.208	16.202
	Dev.MAE 5 Folder	1.10%	1.85%	2.58%	3.84%	3.08%	4.13%
	Dev.MAE 15 Folder	1.02%	0.89%	0.13%	−2.08%	−2.59%	−2.27%
$t_{D}$	*RMSE*	2.285 × 10^−3^	1.727 × 10^−3^	1.724 × 10^−3^	1.501 × 10^−3^	1.505 × 10^−3^	1.499 × 10^−3^
	*R*2	0.38	0.65	0.65	0.73	0.73	0.73
	*MAE*	1.863 × 10^−3^	1.383 × 10^−3^	1.379 × 10^−3^	1.208 × 10^−3^	1.215 × 10^−3^	1.209 × 10^−3^
	Training time	0.453	0.459	34.959	3.909	4.346	8.089
	Dev.MAE 5 Folder	0.12%	2.02%	3.17%	1.37%	0.67%	0.67%
	Dev.MAE 15 Folder	0.92%	−1.02%	0.04%	−2.82%	−3.29%	−3.05%
$t_{D},\;\varepsilon$	*RMSE*	2.277 × 10^−3^	1.485 × 10^−3^	1.478 × 10^−3^	1.172 × 10^−3^	1.173 × 10^−3^	1.172 × 10^−3^
	*R*2	0.38	0.74	0.74	0.84	0.84	0.84
	*MAE*	1.819 × 10^−3^	1.156 × 10^−3^	1.152 × 10^−3^	9.310 × 10^−4^	9.374 × 10^−4^	9.310 × 10^−4^
	Training time	0.561	0.477	26.148	3.549	4.602	12.395
	Dev.MAE 5 Folder	−0.42%	−0.22%	−0.35%	−1.00%	−0.84%	−0.99%
	Dev.MAE 15 Folder	0.20%	0.55%	0.71%	−1.11%	−1.17%	−1.11%
$t_{D},\;\varepsilon ,\;T_{E}$	*RMSE*	2.301 × 10^−3^	1.809 × 10^−3^	1.837 × 10^−3^	1.624 × 10^−3^	1.631 × 10^−3^	1.625 × 10^−3^
	*R*2	0.37	0.61	0.6	0.69	0.68	0.69
	*MAE*	1.881 × 10^−3^	1.453 × 10^−3^	1.473 × 10^−3^	1.301 × 10^−3^	1.309 × 10^−3^	1.302 × 10^−3^
	Training time	0.481	0.393	13.291	2.985	3.759	8.552
	Dev.MAE 5 Folder	−0.56%	1.18%	1.18%	−0.03%	0.34%	0.12%
	Dev.MAE 15 Folder	0.25%	−0.25%	−0.26%	−0.22%	−0.38%	−0.31%
$t_{D},\;\varepsilon ,\;T_{E},\;k$	*RMSE*	2.323 × 10^−3^	1.984 × 10^−3^	1.986 × 10^−3^	1.864 × 10^−3^	1.866 × 10^−3^	1.866 × 10^−3^
	*R*2	0.36	0.53	0.53	0.59	0.59	0.59
	*MAE*	1.913 × 10^−3^	1.628 × 10^−3^	1.631 × 10^−3^	1.516 × 10^−3^	1.517 × 10^−3^	1.517 × 10^−3^
	Training time	0.494	0.368	6.056	2.762	3.132	9.243
	Desv MAE	−0.58%	−0.09%	−0.75%	−14.21%	−13.40%	−14.07%
	Desv MAE	0.22%	−0.18%	−0.57%	0.12%	0.13%	0.05%

**Table A6:** Performance results for defect length predictor models using dataset of 500 sets of values. SVM, MLP and RF when “Contrast Rear” is not contemplated as feature.

		SVM			Multilayer Perceptron	Random Forest
		Cubic	Quadratic	Medium Gaussian		
None	*RMSE*	1.677 × 10^−3^	1.829 × 10^−3^	2.000 × 10^−3^	2.290 × 10^−3^	2.490 × 10^−3^
	*R*2	0.67	0.60	0.53	0.54	0.30
	*MAE*	1.276 × 10^−3^	1.440 × 10^−3^	1.614 × 10^−3^	1.810 × 10^−3^	2.140 × 10^−3^
	Training time	0.221	0.246	0.220	0.177	0.114
	Dev.MAE 5 Folder	4.73%	0.20%	2.51%	−3.31%	0.93%
	Dev.MAE 15 Folder	0.89%	−3.82%	1.77%	−2.76%	−0.47%
$t_{D}$	*RMSE*	1.845 × 10^−3^	1.944 × 10^−3^	2.066 × 10^−3^	2.350 × 10^−3^	2.480 × 10^−3^
	*R*2	0.60	0.55	0.49	0.52	0.29
	*MAE*	1.443 × 10^−3^	1.540 × 10^−3^	1.662 × 10^−3^	1.890 × 10^−3^	2.120 × 10^−3^
	Training time	0.213	0.210	0.214	0.135	0.113
	Dev.MAE 5 Folder	4.05%	8.07%	−3.49%	−4.23%	0.94%
	Dev.MAE 15 Folder	−0.63%	0.14%	−1.28%	−2.65%	−0.94%
$t_{D},\;\varepsilon$	*RMSE*	1.900 × 10^−3^	1.708 × 10^−3^	1.731 × 10^−3^	2.060 × 10^−3^	2.270 × 10^−3^
	*R*2	0.57	0.65	0.64	0.59751354	0.421201
	*MAE*	1.338 × 10^−3^	1.338 × 10^−3^	1.360 × 10^−3^	1.640 × 10^−3^	1.930 × 10^−3^
	Training time	0.297	0.278	0.280	0.118	0.085
	Dev.MAE 5 Folder	1.27%	5.04%	12.06%	6.10%	1.55%
	Dev.MAE 15 Folder	−6.52%	4.62%	6.00%	2.44%	−1.04%
$t_{D},\;\varepsilon ,\;T_{E}$	*RMSE*	1.991 × 10^−3^	2.029 × 10^−3^	2.073 × 10^−3^	2.400 × 10^−3^	2.400 × 10^−3^
	*R*2	0.53	0.51	0.49	0.5	0.34
	*MAE*	1.557 × 10^−3^	1.606 × 10^−3^	1.650 × 10^−3^	1.930 × 10^−3^	2.050 × 10^−3^
	Training time	0.236	0.227	0.219	0.096	0.084
	Dev.MAE 5 Folder	5.04%	3.57%	1.25%	−1.04%	0.49%
	Dev.MAE 15 Folder	−3.19%	0.60%	−0.47%	−2.59%	−0.98%
$t_{D},\;\varepsilon ,\;T_{E},\;k$	*RMSE*	2.296 × 10^−3^	2.333 × 10^−3^	2.151 × 10^−3^	2.520 × 10^−3^	2.340 × 10^−3^
	*R*2	0.37	0.35	0.45	0.45	0.37
	*MAE*	1.793 × 10^−3^	1.834 × 10^−3^	1.722 × 10^−3^	2.040 × 10^−3^	1.980 × 10^−3^
	Training time	0.278	0.262	0.242	0.089	0.080
	Desv MAE	−8.80%	−9.32%	−2.99%	−4.41%	0.51%
	Desv MAE	0.86%	−1.85%	0.38%	−2.45%	−0.51%
